# Pathological convergence of *APP* and *SNCA* deficiency in hippocampal degeneration of young rats

**DOI:** 10.1038/s41419-023-05846-5

**Published:** 2023-05-13

**Authors:** Yajie Wang, Zhikang Miao, Chang Xu, Ying Cai, Yuting Yang, Yue Hu, Mengna Zhao, Yue Shao, Zhiqiang Li, Jincao Chen, Shi Chen, Lianrong Wang

**Affiliations:** 1 Brain Center, Department of Neurosurgery, Ministry of Education Key Laboratory of Combinatorial Biosynthesis and Drug Discovery, Zhongnan Hospital of Wuhan University, School of Pharmaceutical Sciences, Wuhan University, Wuhan, 430071 China; 2 The Research and Application Center of Precision Medicine, The Second Affiliated Hospital of Zhengzhou University, Zhengzhou University, Zhengzhou, 450014 China; 3 Department of Burn and Plastic Surgery, Shenzhen Institute of Translational Medicine, Health Science Center, the First Affiliated Hospital of Shenzhen University, Shenzhen Second People’s Hospital, Shenzhen, 518035 China

**Keywords:** Mechanisms of disease, Neuroscience

## Abstract

The common pathogenesis of Alzheimer’s disease (AD) and Parkinson’s disease (PD) has been supported by biochemical, genetic and molecular evidence. Mitochondrial dysfunction is considered to be the common pathology in early AD and PD. The physiological regulation of APP and α-synuclein on mitochondria remains unclear, let alone whether they share common regulatory mechanisms affecting the development of neurodegenerative diseases. By studying gene knockout rats, the commonality of physiological APP and α-synuclein in maintaining mitochondrial function through calcium homeostasis regulation was revealed, which was critical in inhibiting hippocampal degeneration in young rats. APP and α-synuclein both control hippocampal mitochondrial calcium intake and outflow. In the mitochondrial calcium influx regulation, APP and α-synuclein are located on the mitochondrial-associated endoplasmic reticulum membrane (MAM) and converge to regulate the IP_3_R1-Grp75-VDAC2 axis. Mitochondrial calcium outflow is redundantly promoted by both α-synuclein and APP. Loss of APP or SNCA leads to mitochondrial calcium overload, thus enhancing aerobic respiration and ER stress, and ultimately causing excessive apoptosis in the hippocampus and spatial memory impairment in young rats. Based on this study, we believe that the physiological function impairment of APP and SNCA is the early core pathology to induce mitochondrial dysfunction at the early stage of AD and PD, while the IP_3_R1-Grp75-VDAC2 axis might be the common drug target of these two diseases.

## Introduction

Alzheimer’s disease (AD) and Parkinson’s disease (PD) are the two most common and widely occurring neurodegenerative diseases at present. They share similar pathological characteristics, such as mitochondrial dysfunction in the early stage [1] and physiological toxicity caused by abnormal protein aggregates in the middle stage accompanied by mild cognitive impairment [2]. Although the copathogenesis of AD and PD has been explored from biochemical [3], genetic [4] and molecular [5, 6] perspectives, the details are still not clear. Abnormal deposition occurs in the middle stage of the disease, when the types of sediment, physiological toxicity effects and brain regions involved are different, and it is difficult to find a general rule. Therefore, the middle stage is not suitable for studying copathogenesis.

Mitochondrial dysfunction is the common pathological feature of AD and PD in the early stage [7], when abnormal aggregates have not yet been produced. The endogenous functional impairment of *APP* and *SNCA*, encoding genes of Aβ and α-synuclein, respectively, might be the reason for mitochondrial dysfunction. However, most studies thus far have established research models by overexpressing mutated genes [1], covering the phenotype caused by endogenous *APP* and *SNCA* functions. The mitochondrial regulation of these genes has similarities. First, both are located in the mitochondria. Mutated APP is positioned through mitochondrial double membranes by “N-terminal to mitochondrial matrix—C-terminal to cytoplasm” [8], while α-synuclein binds to the mitochondrial outer membrane [9]. Second, overexpression of *APP* or *SNCA* leads to abnormal mitochondrial morphology and function. Overexpression of wild-type *SNCA* leads to a special phenotype of mitochondrial enlargement and mitochondrial crystal vacuolation in mouse hypothalamic tumor cells [10]. Overexpressed wild-type *APP* causes mitochondrial fragmentation in human neuroblastoma cells and blocks ATP synthesis [11]. Third, APP and α-synuclein were found in endoplasmic reticulum—mitochondria coupling (MAM). In mouse brain and cell lines, APP is distributed in the MAM fraction and spliced to obtain C99, which is thought to help maintain the number of MAM [12, 13]. In the SH-SY5Y cell line, overexpressed α-synuclein interferes with the VAPB-PTPIP51 tether in the MAM structure, blocking calcium flow from the ER to mitochondria, and thereby reducing ATP synthesis [14]. It is still unclear whether APP and α-synuclein have commonality in MAM-mediated mitochondrial regulation of the central nervous system, which could be the copathology in early-AD and PD. To this end, we studied and compared the regulation of APP and α-synuclein on MAM in the hippocampus of gene knockout rats at a young age.

## Results

### Both *APP* and *SNCA* deficiency lead to hippocampal degeneration

**Fig. 1:**
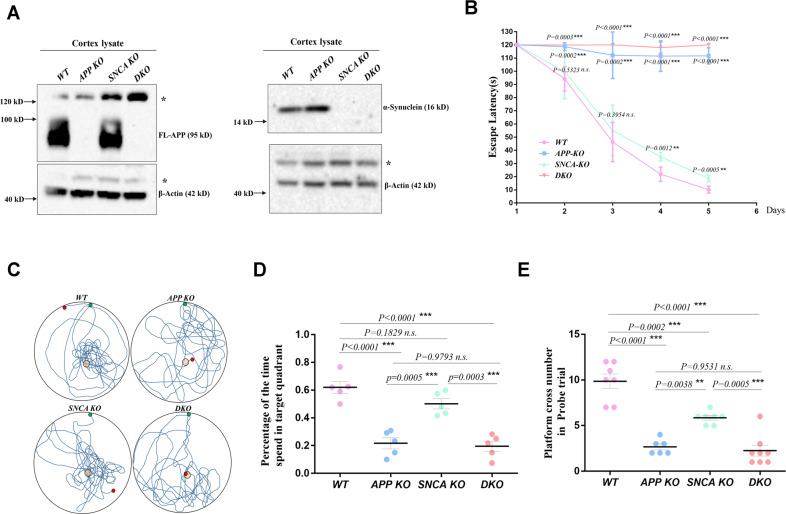
*APP* and *SNCA* deficiency lead to spatial memory impairment in young rats. **A** The protein levels of APP and SNCA in cortical lysates from *WT*, *APP KO* (*APP*-deficient), *SNCA KO* (*SNCA*-deficient) and *DKO* (*APP*- and *SNCA*-deficient) rats were measured by western blotting. The molecular weight of each protein is provided on the right. FL-APP was completely absent in the *APP KO* rat cortex, and α-synuclein was completely absent in the *SNCA KO* rat cortex. *DKO* rats lacked both *APP* and *SNCA* proteins. **B–E** MWM test with 3-month-old male rats. Three independent experiments including at least 5 rats per genotype were performed. The escape latency curve during the 5-day training period is shown in (**B**). Data from each day compared to *WT* rats were analyzed by one-way ANOVA. **p < 0.01 and ***p < 0.001 indicate significant differences, and *n.s*. indicates no significance. Representative tracks of individual rats in the probe trails are shown in (**C**). The percentage of the time spent in the target quadrant in the probe trial is shown in (**D**). The number of platform crossings in the probe trial is shown in (**E**). The data of individual rats are indicated by dots, and the mean ± SEM for each genotype is presented in (**D**) and (**E**). One-way ANOVA was used. *p < 0.05, **p < 0.01, and ***p < 0.001 indicate significant differences, and *n.s*. indicates no significance.

To explore the physiological functions of *APP* and *SNCA*, *APP KO* and *SNCA KO* rats were generated by TALEN (Fig. S1A), and *APP-SNCA DKO* rats were obtained through hybridization. Full-length APP (FL-APP) and α-synuclein were completely absent in brain tissue samples of corresponding genotypes (Fig. 1A), proving that these gene knockout models can be used for subsequent study.

The Morris water Maze (MWM) experiment was used to assess the spatial memory function [15] of the rat hippocampus (Fig. 1B–E). During the 5-day continuous training, the time for *WT* rats to find the escaping platform was gradually shortened, which showed normal learning abilities. On day 4 and day 5 of training, the performance of *SNCA KO* rats was slightly worse than that of *WT* rats. *APP KO* and *DKO* rats did not make any progress during the 5-day training and could not remember the location of the escaping platform, suggesting that these rats had obvious learning impairment (Fig. 1B). On the probe day of the MWM, *WT* rats spent 62.1% of the total time exploring the target quadrant, and crossed the platform position >9.86 times within 2 min (Fig. 1C–E), indicating that *WT* rats could accurately remember the platform position with their normal memory formation, storage and extraction abilities. *SNCA KO* rats stayed in the target quadrant for 50.2% of the total time, and crossed the platform position ~5.86 times. Compared with *WT* rats, *SNCA KO* rats showed confusion about the exact position of the platform, suggesting that *SNCA* deficiency causes mild spatial memory impairment in these rats. The probe time of *APP KO* rats in the target quadrant accounted for 21.7%, with the number of crossings not exceeding 2.67. *DKO* rats showed similar results to *APP KO* rats, with an average of ~2.25 crossing platform positions and 19.5% detection time in the target quadrant. The swimming speed and movement distance of genetically defective rats showed no significant difference from those of *WT* rats (Fig. S1B, C), suggesting that the genetic defect does not affect the motor ability of young animals. These data showed that the exploration behavior of *APP KO* and *DKO* rats was random and had no preference, indicating that these rats could not learn to escape during the 5-day training, and their cognitive ability was severely impaired.

**Fig. 2:**
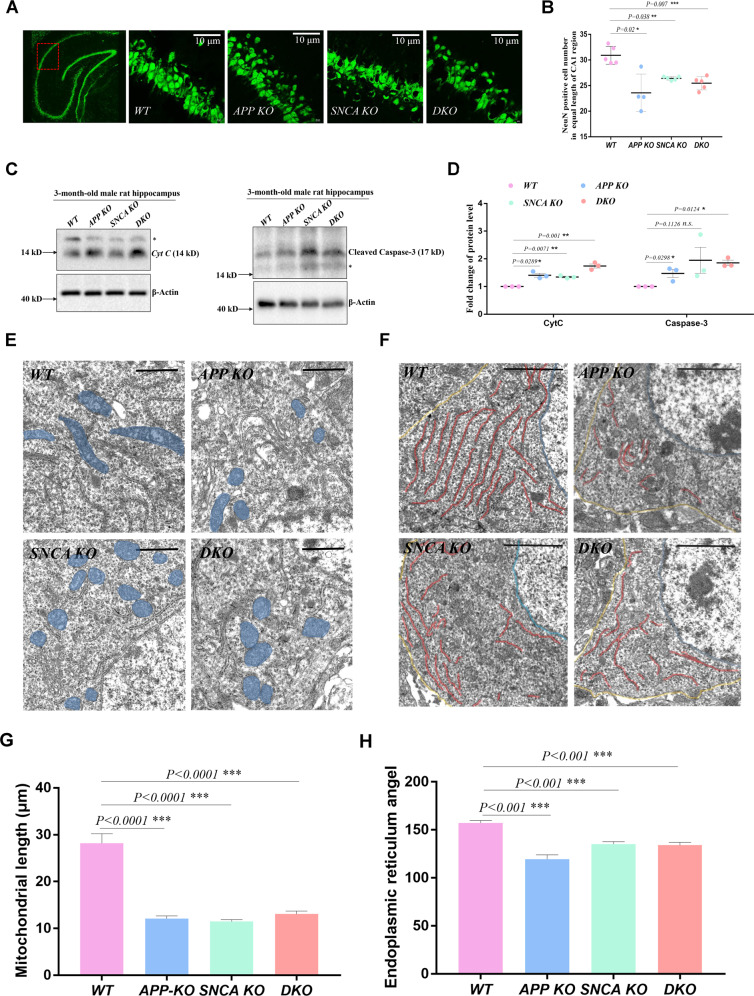
*APP* and *SNCA* deficiency lead to hippocampal degeneration. **A**, **B** Immunostaining of brain slices performed with a NeuN antibody. Coronal slices from each rat were prepared to include the bilateral hippocampus. Two slices per rat were used for NeuN immunostaining. Under 60× objective vision, a complete field of two red squares (indicated by the red box) in the CA1 region of the unilateral hippocampus was photographed, and the NeuN-positive neurons in each field were counted. Eight fields from each rat were assayed, and the average value of an individual rat is indicated by a single dot in (**B**). Three independent experiments with *WT* (*n* = 5), *APP KO* (*n* = 4), *SNCA KO* (*n* = 5) and *DKO* (*n* = 5) rats were executed with 3-month-old male rats. The data of individual rats are shown as the mean ± SEM. *T*-tests were performed. *p < 0.05, **p < 0.01, and ***p < 0.001 indicate significant differences. **C**, **D** Western blotting of *Cyt C* and cleaved Caspase-3 in the rat hippocampi of the indicated genotypes. The stray band is indicated by the asterisk. A single rat per genotype was detected in one experiment, as shown in (**C**). Three independent experiments of three 3-month-old male rats per genotype were performed. The data are presented as the mean ± SEM in (**D**) with the dots indicating data from individual rats. *T*-tests were performed. *p < 0.05 and **p < 0.01 indicate significant differences, and *n.s*. indicates no significance. **E**, **F** Representative TEM images of pyramidal neurons in hippocampal slices from 3-month-old male rats. Mitochondria and the ER network in the neuronal soma are shaded blue and red, respectively. The yellow lines indicate the PM, and the blue lines indicate the nuclear envelope in the neuronal soma in (**F**). Scale bar = 10 μm in (**E**) and 20 μm in (**F**). **G** Mitochondrial morphology assay. The length between the longest two ends of each mitochondrial particle in TEM photos was measured. The data are presented as the mean ± SEM with at least five photos of each genotype. *T*-tests were used. ***p < 0.001 indicates a significant difference. **H** ER morphology assay. The bending angle of the ER lamella was measured in TEM photos. The data are presented as the mean ± SEM with at least five photos of each genotype. *T*-tests were used. ***p < 0.001 indicates a significant difference.

To further confirm that the spatial memory impairment of gene-deficient rats is caused by hippocampal injury, the lamellar structure of neurons in the CA1 region was observed by anti-NeuN antibody staining. The pyramidal neurons in the CA1 region of *WT* rats were arranged in layers, while some local neuron vacancies appeared in the same CA1 region of gene deletion rats (Fig. 2A). We calculated the number of NeuN-positive neurons in the equal-length CA1 region, which was significantly less in *APP KO*, *SNCA KO*, and *DKO* rats (Fig. 2B). Nissl’s staining of the rat hippocampus showed similar results (Fig. S1D, S1E). Considering that loss of neurons in the CA1 region may be caused by abnormal apoptosis, we examined the levels of *Cyt C* and cleaved caspase-3 in hippocampal lysates of 3-month-old male rats and found that they were upregulated in gene-deficient rats (Fig. 2C, D). These results suggest that excessive apoptosis damaged the hippocampal structure of *APP KO* and *SNCA KO* rats.

Changes in hippocampal structure and impairment in spatial memory in young rats suggested that *APP* and *SNCA* deficiency caused hippocampal degeneration. In other words, *APP* and α-synuclein are necessary in maintaining normal hippocampal physiology. By comparing the performance of rats with different genotypes in the water maze tests, we also found that the spatial memory of *APP KO* rats was worse than that of *SNCA KO* rats, while *DKO* rats were similar to *APP KO* rats. This suggests that the physiological APP is more critical than α-synuclein in maintaining hippocampal structure and function, and the two may belong to the same regulatory mechanism.

### Mitochondrial degradation and ER stress in *APP-* and *SNCA-*deficient hippocampi

*Cyt C* is normally stored in the intermembrane space of mitochondrial cristae and is released into the cytoplasm when the mitochondrial-dependent apoptotic pathway is activated [16], initiating irreversible programmed cell death. The upregulation of *Cyt C* suggests that *APP KO* and *SNCA KO* hippocampal mitochondria might be abnormal (Fig. 2C, D). Therefore, the mitochondrial morphology of hippocampal pyramidal neurons was observed through transmission electron microscopy (TEM). In *WT* neuron soma, mitochondria were rod-shaped, while those in mutant neurons were mostly spherical (Fig. 2E) because the length between the farthest ends of mitochondrial granules in gene knockout neurons were ~50% shorter, which was 28.19 μm on average in *WT* neurons, but 12.13 μm in *APP KO*, 11.47 μm in *SNCA KO*, and 13.07 μm in *DKO* neurons (Fig. 2G). Endoplasmic reticulum (ER) morphology was also observed. Its network in *WT* neurons was arranged in parallel, but appeared curled and shortened in mutant neurons with their uniform intracellular distribution abolished (Fig. 2F), as the average bending angle of the ER lamella was larger in *WT* neurons than in mutants (Fig. 2H). These results suggest that *APP* and *SNCA* deficiency may induce mitochondrial and ER dysfunction in hippocampal neurons.

**Fig. 3:**
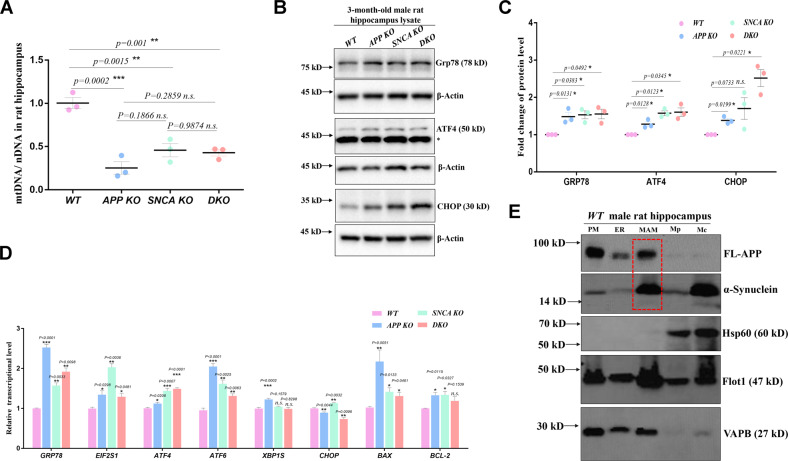
Mitochondrial degradation and ER stress in *APP-* and *SNCA-*deficient hippocampi. **A** Mitochondrial content was assessed by determining the mtDNA/nDNA ratio through qPCR. Three independent experiments of three 3-month-old male rats per genotype were performed. The individual data are indicated with dots. The data for each genotype are presented as the mean ± SEM. One-way ANOVA was used for statistical analysis, and differences were indicated by asterisks. **p < 0.01 and ***p < 0.001 indicate significant differences, and *n.s*. indicates no significance. **B**, **C** The expression of ER stress-related proteins in rat hippocampal lysates was measured. The stray band is indicated by an asterisk. Three independent experiments of 3-month-old male rats per genotype were performed. Each experiment detected one rat from different genotype groups at once as shown in (**B**). Quantification is presented as the mean ± SEM of the data from three rats per genotype in (**C**). *T*-tests were used. *p < 0.05 indicates a significant difference, and *n.s*. indicates no significance. **D** Transcription levels of ER stress**-**related genes were measured by RT–qPCR. RNA was isolated from hippocampal tissues of 3-month-old male rats. Three independent experiments with five rats per genotype were performed. The data are shown in the bar chart as the mean ± SEM. *T*-tests were performed. *p < 0.05, **p < 0.01, and ***p < 0.001 indicate significant differences, and *n.s*. indicates no significance. **E** Subcellular fractions were probed with the indicated antibodies. A total of 15 μg protein from each fraction was loaded per lane. PM plasma membrane, Mc crude mitochondrial fraction, Mp pure mitochondrial fraction. The red box indicates the FL-APP and α-synuclein protein bands in the MAM fraction. Hippocampal tissues from six male rats of 3-month-old were mixed and used for component separation.

To confirm mitochondrial and ER abnormalities, we assayed the mitochondrial fission/fusion balance and ER stress related protein levels in hippocampal lysates. The fission regulators short-OPA1 [17] and Drp1 [18] showed an increasing trend in mutant hippocampal lysates (Fig. S1F,-G). We then assayed the mitochondrial content in the hippocampus through qPCR [19]. The mtDNA/nDNA ratios of *APP KO*, *SNCA KO* and *DKO* hippocampi were 25.3%, 45.5% and 42.6% of *WT*, respectively (Fig. 3A), indicating decreased mitochondrial content in the tissue. Furthermore, upregulation of LC3-II suggested that mitophagy may have been activated [20], although the LC3-II/LC3-I ratio was not significantly different between mutant and *WT* rats in the hippocampus (Fig. S2A, B). These data suggest that *APP* or *SNCA* depletion leads to increased mitochondrial fission, enhanced mitophagy and decreased mitochondrial content, suggesting excessive mitochondrial degradation in *APP*- and *SNCA*-deficient hippocampi. Grp78 is the upstream regulator of the ER stress-activated pathway [21], which was upregulated at both the protein level (Fig. 3B, C) and transcriptional level (Fig. 3D) in the hippocampi of mutant rats. The downstream regulators ATF4 and CHOP of ER stress were upregulated (Fig. 3B, C), which transcriptionally activated the apoptosis-related genes *BAX* and *BCL-2* in the mutant hippocampus (Fig. 3D), suggesting activation of the PERK pathway [22]. These data suggest that loss of either *APP* or *SNCA* results in ER stress and activation of the downstream response pathway.

The physiological states of mitochondria and ER in gene-deficient hippocampi were both changed, suggesting that the functional coordination between mitochondria and ER through the mitochondrial-associated ER membrane (MAM) might be abnormal in *APP KO* and *SNCA KO* hippocampi.

### MAM-localized APP and α-synuclein maintain ER-mitochondrial calcium homeostasis

Different groups have found that APP and α-synuclein localize to MAMs in different cell or animal models [12–14]. To determine whether endogenous APP and α-synuclein were located in the MAM of rat hippocampi, plasma membrane (PM), ER, MAM, and crude and pure mitochondrial (Mc, Mp) components were purified by density gradient centrifugation [23]. Markers were used to determine the purity of these subcellular fractions, such as VAPB for ER, Hsp60 for mitochondria and Flot1 for lipid rafts on the cellular membrane, especially the MAM. The abundance of FL-APP was high in the PM, ER and MAM, and α-synuclein was mainly distributed in the MAM and crude mitochondria (Fig. 3E). Our results were consistent with previous views that FL-APP and α-synuclein are located in the MAM. The MAM structure is maintained by tethers such as Mfn1/2 [24], VAPB-PTPIP51 [25], Fis1-Bap31 [26] and IP_3_Rs-Grp75-VDAC1 [27]. Compared with *WT* hippocampi, levels of certain tether proteins were altered in *APP*- and *SNCA*-deficient hippocampi (Fig. S2C, D), implying that MAM-located FL-APP and α-synuclein may contribute to its biological functions.

The MAM matches ER energy consumption with mitochondrial energy supply through calcium signaling [28]. In general, calcium released from the ER flows into the mitochondrial matrix through the MAM structure to regulate energy metabolism. If excessive calcium is released from the ER through the MAM structure to enter the mitochondria, mitochondrial calcium upregulation and ER lumen calcium depletion would both occur. At this point, the cell triggers a store-operate calcium entry (SOCE) process that couples the ER and plasma membrane via calcium channels, allowing extracellular calcium to flow into the ER lumen for compensation [29]. ORAI 1 is the coupling calcium channel on the ER membrane during SOCE and was found to be increased in *APP KO*, *SNCA KO* and *DKO* hippocampal lysates (Fig. S2E, F), suggesting that the SOCE process may be triggered by ER calcium depletion in these gene-defective hippocampi.

**Fig. 4:**
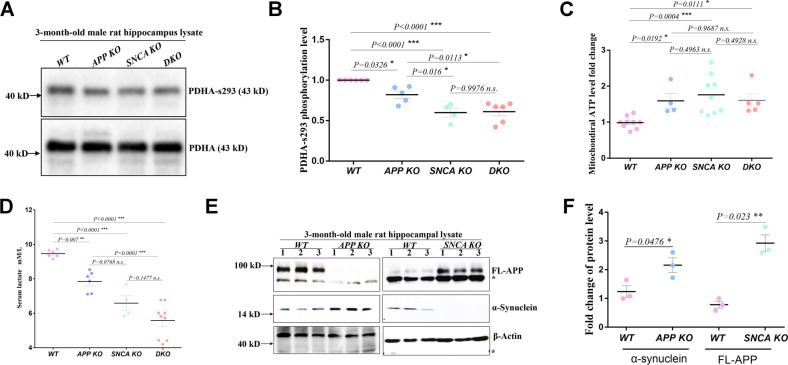
MAM-located APP and α-synuclein maintain ER-mitochondrial calcium homeostasis. **A**, **B** Western blot analysis of PDHA^s293^ (PDH phosphorylated at Ser293 of the E1-α subunit) and total PDHA protein levels in hippocampal lysates from rats of the indicated genotype. Three independent experiments of (**A**) with at least four male rats of 3-months old per genotype were used for quantification. The PDHA^s293^/PDHA level of individual rats is indicated by a dot in (**B**). The mean ± SEM for each genotype is presented, including *WT* (*n* = 6), *APP KO* (*n* = 5), *SNCA KO* (*n* = 4) and *DKO* (*n* = 6). One-way ANOVA was used for statistical analysis, and differences are indicated by asterisks. *p < 0.05 and ***p < 0.001 indicate significant differences, and *n.s*. indicates no significance. **C** ATP levels in mitochondria isolated from the rat hippocampi of the indicated genotype. At least four male rats of 3-month-old per genotype were studied in three independent experiments. The data of individual rats are indicated by dots, and the mean ± SEM for each genotype is presented. One-way ANOVA was used for statistical analysis, and differences are indicated by asterisks. *p < 0.05 and ***p < 0.001 indicate significant differences, and *n.s*. indicates no significance. **D** Serum lactate levels of rats with the indicated genotype. At least four male rats of 3-month-old per genotype were assayed. The data of individual rats are indicated by dots, and the mean ± SEM for each genotype is presented. One-way ANOVA was used for statistical analysis, and differences are indicated by asterisks **p < 0.01 and ***p < 0.001 indicate significant differences, and *n.s*. indicates no significance. **E**, **F** The protein levels of FL-APP and α-synuclein in the hippocampi of 3-month-old male knockout rats were measured in *SNCA KO* and *APP KO* rats by western blotting in (**E**). Tissue samples were collected from three rats of each genotype. The asterisk indicates a stray band. Quantification is shown in (**F**) with the mean ± SEM, and each dot represents the data from an individual rat. *T*-tests were used. *p < 0.05 and ***p < 0.001 indicate significant differences.

Excessive calcium flux into the mitochondrial matrix could enhance the citric acid cycle (TCA cycle) because the activities of three key enzymes of this metabolic pathway are sensitive to the matrix calcium concentration, including pyruvate dehydrogenase [30]. Thus, the phosphorylation level of pyruvate dehydrogenase (PDHA^s293^) in the hippocampus lysates was detected, which had been proven to be sensitive to the matrix calcium concentration and was significantly lower in the *APP KO*, *SNCA KO* and *DKO* hippocampus, at 82.1%, 59.9% and 61.1% of the levels in *WT* rats, respectively (Fig. 4A, B). These data suggested that *APP KO*, *SNCA KO* and *DKO* rats have higher concentrations of mitochondrial matrix calcium in the hippocampus. This is supported by the fact that mitochondrial ATP levels increased in the mutant hippocampus, which were 1.60 (*APP KO*), 1.76 (*SNCA KO*) and 1.61 (*DKO*) times that in *WT* mitochondria (Fig. 4C), and serum lactic acid was reduced in *APP KO* (7.85 mM/L), *SNCA KO* (6.59 mM/L) and *DKO* (5.57 mM/L) rats compared to *WT* rats (9.47 mM/L) (Fig. 4D). These findings confirmed that enhanced aerobic respiration in *APP* and *SNCA KO* hippocampi was caused by elevated mitochondrial calcium levels.

These results showed that endogenous FL-APP and α-synuclein were located on MAMs and were involved in maintaining ER and mitochondrial calcium homeostasis. Loss of either protein leads to ER stress and enhanced respiration in the rat hippocampus due to the disruption of MAM-mediated calcium homeostasis.

### APP and α-synuclein cooperatively control MAM-mediated mitochondrial calcium inflow

To explore the regulatory relevance of *APP* and *SNCA* on mitochondrial calcium homeostasis, we compared their protein levels in hippocampal lysates between mutant and *WT* rats. In *APP KO* hippocampi, α-synuclein expression was upregulated ~1.74-fold, while FL-APP protein levels were elevated ~3.73-fold in *SNCA KO* hippocampi (Fig. 4E-F). These data showed dose compensation between FL-APP and α-synuclein, suggesting their functional correlation.

**Fig. 5:**
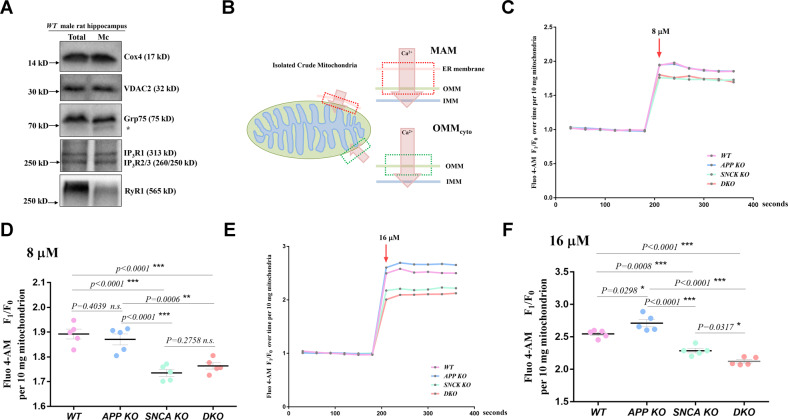
APP and α-synuclein cooperatively control MAM calcium flow. **A** The calcium channels in the crude mitochondrial fraction isolated from the hippocampi of male *WT* rats were measured by western blotting. Total total homogenate, Mc crude mitochondria. The molecular weight of each protein is provided on the right. The stray band is indicated by an asterisk. **B** Diagram of the two calcium flow paths into isolated crude mitochondria. The arrows indicate the direction of calcium flux. The red box indicates calcium flow through the MAM. The green box indicates calcium flow through the OMM_cyto_. Calcium flowing into the mitochondrial matrix through the MAM needs to pass through three layers, including the ER membrane, OMM and inner mitochondrial membrane (IMM), while calcium flowing through the OMM_cyto_ needs to cross only two layers, the OMM and IMM. A higher concentration of calcium should flow through the MAM than through the OMM_cyto_ because the calcium concentration in the ER lumen is usually 1000 times higher than that in the cytoplasm [28]. **C** Representative fluorescence traces showing mitochondrial calcium uptake after 8 μM calcium stimulation. Ten milligrams of isolated crude hippocampal mitochondria from 3-month-old male rats were loaded with Fluo 4-AM and used for the assay. The fluorescence was measured 6 times every 30 s before and after stimulation buffer was added. The average mitochondrial fluorescence count before stimulation was used as an internal reference (F_0_). The fluorescence fold change relative to F_0_ (F_1_/F_0_) was used for normalization to assess calcium uptake. The F_1_/F_0_ value is plotted on the y-axis, and the detection time is plotted on the *x*-axis. The red arrow indicates the addition of 8 μM CaCl_2_ buffer for stimulation. **D** Three independent experiments including at least five rats per genotype were performed as described in (**C**). The average F_1_/F_0_ values after 8 μM calcium stimulation of hippocampal mitochondria from individual rats are indicated by dots. The mean ± SEM for each genotype is presented. One-way ANOVA was used for statistical analysis, and differences are indicated by asterisks. **p < 0.01 and ***p < 0.001 indicate significant differences, and *n.s*. indicates no significance. **E**, **F** Similar experiments were performed with 16 μM calcium stimulation. Representative fluorescence traces are shown in (**E**), and quantitative data for the four genotypes are presented in (**F**). The F_1_/F_0_ values of individual rats are indicated by dots. The mean ± SEM for each genotype is presented. One-way ANOVA was used for statistical analysis, and differences are indicated by asterisks. *p < 0.05 and ***p < 0.001 indicate significant differences.

MAM is a tightly coupled structure of the ER and mitochondrial outer membrane (OMM) and is usually attached to the OMM and precipitated by centrifugation together with isolated mitochondria [23], which inspired us to study the MAM-mediated regulation of calcium flow by using crude mitochondria from the rat hippocampus. Various calcium channels of the ER and OMM are enriched in MAM [31], and we detected the ER calcium channels IP_3_Rs and RyR1 in isolated crude mitochondria to confirm the attachment of MAM (Fig. 5A). The OMM calcium channels VDAC2 and Grp75, which tether IP_3_Rs and VDAC1 [32], were observed. Therefore, we believe that isolated crude mitochondria can be used to study the effect of MAM on mitochondrial calcium intake.

Intracellular mitochondria respond to calcium fluctuations through either the mitochondria-cytoplasm interface (OMM_cyto_) or the mitochondria-ER interface (MAM), which differ in the number of layers and ion flow concentration (Fig. 5B). The concentration of calcium flow via the OMM_cyto_ should be lower than that via the MAM, where high-calcium “hot spots” >10 μM can be formed instantly [28]. We loaded the hippocampal isolated crude mitochondria with the calcium-sensitive dye Fluo 4-AM and monitored their fluorescence change (F_1_/F_0_) in response to extracellular stimulation with 8 μM and 16 μM calcium, lower and higher than10 μM, respectively (Fig. 5C–F). The fluorescence counts increased after stimulation, as mitochondria took up calcium from the surrounding fluid (Fig. 5C, E). *WT* mitochondrial fluorescence increased 1.89-fold after 8 μM calcium stimulation (Fig. 5C, D), suggesting that the net calcium intake was equivalent to 89% of its resting calcium pool. The F_1_/F_0_ of *APP KO* mitochondria was 1.87, similar to that of *WT* mitochondria, indicating that *APP* deficiency did not affect mitochondrial calcium uptake after 8 μM stimulation. However, the F_1_/F_0_ ratio of *SNCA KO* and *DKO* mitochondria was significantly decreased to 1.74 and 1.76, respectively, suggesting that *SNCA* deletion leads to a decrease in mitochondrial calcium uptake. Since FL-APP is rarely localized, while α-synuclein is localized to the OMM, it is reasonable that α-synuclein facilitates the absorption of mitochondrial calcium through the OMM under calcium stimulation of <10 μM, rather than FL-APP.

We then stimulated hippocampal mitochondria with 16 μM calcium (Fig. 5E-F). The fluorescence fold change of *WT* mitochondria after stimulation was 2.55 times relative to its resting fluorescence. The *APP KO* mitochondria took up more calcium than the *WT* mitochondria, as the F_1_/F_0_ value was 2.71. Since FL-APP is primarily localized to the MAM but not the OMM_,_ we believe that FL-APP inhibits calcium flow passing through the ER membrane into the MAM under stimulation. The F_1_/F_0_ index of *SNCA KO* mitochondria was 2.29, which was lower than that of *WT* mitochondria, in line with the role of α-synuclein in promoting calcium ions to enter mitochondria through the OMM either at MAM or OMM_cyto_. The *DKO* mitochondrial F_1_/F_0_ index was 2.12, which was lower than that of *WT* mitochondria, indicating that *APP* deficiency-induced excess calcium flow into mitochondria through the MAM was dependent on α-synuclein. In addition, calcium uptake by *DKO* mitochondria was less than that of *SNCA KO* mitochondria, suggesting a feebly promoting role of APP on calcium entering mitochondria through the OMM at the MAM region.

Based on these data, we believe that APP bidirectionally regulates MAM calcium flow, inhibits excessive calcium release from the ER lumen to the MAM region, and synergistically promotes calcium flow from the MAM region to the mitochondrial matrix with α-synuclein, but the latter plays a more decisive role than APP during the calcium entry process.

### The regulation of APP and α-synuclein on MAM-mediated mitochondrial intake converges on the IP_3_R1-Grp75-VDAC2 axis

**Fig. 6:**
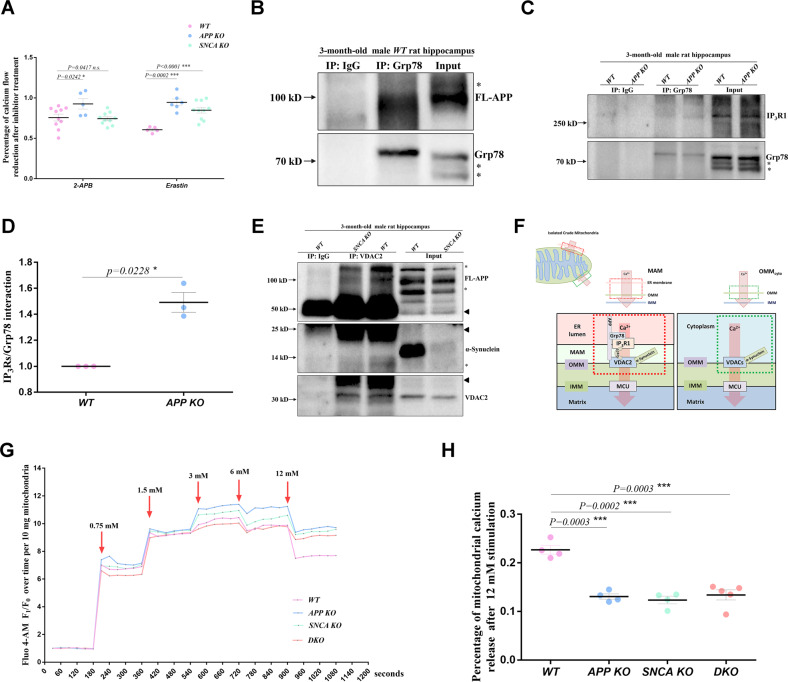
APP and α-synuclein converge on the IP_3_R1-Grp75-VDAC2 axis. **A** The sensitivity of hippocampal mitochondria to the calcium channel antagonists 2-APB and erastin was assessed. Ten milligrams of isolated hippocampal mitochondria from 3-month-old male rats were loaded with Fluo 4-AM and then incubated with 200 μM 2-APB or 20 μM erastin. With 16 μM calcium stimulation, mitochondria in which calcium channels were inhibited showed a smaller increase in fluorescence count than untreated controls. The percentages of net fluorescence increase in the antagonist-treated mitochondria relative to its untreated controls were determined. Three independent experiments including at least five rats per genotype were performed. Data from individual rats are indicated by dots, and the mean ± SEM for each genotype is presented. *T*-tests were used. *p < 0.05 and ***p < 0.001 indicate significant differences, *n.s*. indicates no significance. **B** Coimmunoprecipitation of Grp78 and FL-APP in hippocampal lysates from 3-month-old *WT* male rats. Anti-Grp78 antibody was used for immunoprecipitation, and Grp78 protein pulled down from the lysate was used as the loading control. Anti-APP antibody was used for western blotting. The stray bands are indicated by asterisks. **C** Comparison of the IP_3_R1-Grp78 interaction in the hippocampi of 3-month-old *WT* and *APP KO* male rats. An anti-Grp78 antibody was used for immunoprecipitation, and Grp78 protein pulled down from the lysate was used as the loading control. IP_3_R1 levels were measured by western blotting, and the stray bands are indicated by asterisks. **D** Three independent experiments in (**C**) including three rats per genotype were used for quantification. The mean ± SEM for each genotype is presented. *T*-test was used for statistical analysis, *p < 0.05 indicates a significant difference. **E** Interactions of VDAC2 with FL-APP and α-synuclein in the hippocampi of 3-month-old *WT* and *SNCA KO* male rats. A VDAC2 antibody was used for immunoprecipitation, and VDAC2 protein pulled down from the lysate was used as the loading control. FL-APP and α-synuclein were detected through western blotting. Immunoprecipitation was performed in *SNCA KO* hippocampal lysates as a negative control. The stray bands are indicated by asterisks. The arrowheads indicate the IgG heavy/light chain. Three independent experiments including 3 rats per genotype were performed. **F** Schematic diagram of the mechanism by which APP and α-synuclein regulate calcium flow via the IP_3_R1-Grp75-VDAC2 axis in MAMs. FL-APP interacts with Grp78 to disrupt the binding of Grp78 to IP_3_R1, prohibiting calcium flow released from the ER lumen into the MAM. Both FL-APP and α-synuclein interact with VDAC2 to promote calcium flow into the matrix. At OMM_cyto_, only α-synuclein binds to VDAC channels to regulate calcium flux. **G** Representative fluorescence traces showing mitochondrial calcium uptake after a series of extra calcium stimulations ranging from 0.75 mM to 12 mM. Ten milligrams of crude hippocampal mitochondria isolated from 3-month-old male rats and loaded with Fluo4-AM were used for the assay. The fluorescence count was measured every 30 s and six times before and after each stimulation. The average fluorescence count of unstimulated mitochondria of each genotype was used as an internal reference (F_0_), and all fluorescence measurements were divided by F_0_ for normalization (F_1_/F_0_). The F_1_/F_0_ value is plotted on the y-axis, and the detection time is plotted on the *x*-axis. The red arrow indicates the addition of CaCl_2_ buffer for stimulation. **H** Quantification of mitochondrial calcium release after 12 mM extra calcium stimulation. Three independent experiments including at least four male rats per genotype at 3 months old were performed as described in (**G**). The percentage of net reduction in the average F_1_/F_0_ value after 12 mM calcium stimulation relative to the plateau level of 3–6 mM calcium stimulation was determined. The data of individual rats are indicated by dots, and the mean ± SEM for each genotype is presented. *T*-tests were used for statistical analysis, and differences are indicated by asterisks. ***p < 0.001 indicates significant differences.

To determine the calcium channels that are regulated by FL-APP and α-synuclein, we incubated isolated hippocampal mitochondria with antagonists (Fig. 6A, Fig. S3A, B). 2-Aminoethyl diphenylborinate (2-APB) is a specific antagonist of IP_3_Rs channels [33] and was used to incubate mitochondria before 16 μM calcium stimulation. For quantification, we set the net fluorescence increase of untreated mitochondria after stimulation as 100%. The net fluorescence increase of 2-APB-treated *WT* mitochondria was 70.3% of the untreated control (Fig. S3A), indicating that 2-APB inhibited the IP_3_Rs channels to reduce calcium uptake by *WT* mitochondria and that *WT* mitochondria were sensitive to this antagonist. *APP KO* mitochondria appeared insensitive to 2-APB, as the calcium uptake by 2-APB-treated *APP KO* mitochondria was 92.7% of the untreated *APP KO* controls (Fig. 6A). The tolerance of *APP KO* mitochondria to 2-APB indicates that *APP* deficiency disrupts the normal conduction of IP_3_Rs channels. Calcium uptake by 2-APB-treated *SNCA KO* mitochondria was 74.3% of that by untreated *SNCA KO* controls, similar to *WT* mitochondria, indicating that α-synuclein does not affect IP_3_Rs channels.

Azumolene, erastin and NSC15364 were used to specifically perturb the RyRs [34], VDAC2/3 [35] and VDAC1 [36] channels, respectively (Fig. 6A, Fig. S3A, B). The sensitivity of gene-deficient mitochondria to azumolene and NSC15364 was similar to that of *WT* mitochondria (Fig. S3B), indicating that *APP* and *SNCA* deficiency does not affect the calcium permeability of RyRs and VDAC1 in MAM. *APP KO* (94.5% of untreated controls) and *SNCA KO* (84.8% of untreated controls) mitochondria appeared less sensitive to erastin than *WT* mitochondria (60.7% of untreated controls) (Fig. 6A), suggesting that the calcium permeability of VDAC2/3 is affected by APP and α-synuclein. As VDAC3 is an ROS sensor but not a calcium regulator [37], we believe that APP and α-synuclein affect VDAC2 at MAMs.

We then used an IP_3_Rs antibody for immunoprecipitation in hippocampal tissue lysates and probed with an APP antibody by western blotting. No interaction was found between FL-APP and IP_3_Rs (Fig. S3C), indicating that FL-APP may regulate IP_3_Rs channels through mediators. Grp78 has been shown to interact with IP_3_R1 channels and promote channel opening and ER calcium release [38]. Furthermore, APP binds to Grp78 on the ER [39]. Therefore, we confirmed the APP-Grp78 interaction through coimmunoprecipitation (Fig. 6B) and assessed whether the Grp78-IP_3_R1 interaction was affected by *APP* depletion. The Grp78-IP_3_R1 interaction was enhanced in the *APP KO* hippocampus, as ~1.49 times more IP_3_R1 was coimmunoprecipitated with Grp78 antibody from *APP KO* rat hippocampal lysates than from *WT* lysates (Fig. 6C, D). These results indicated that FL-APP regulates IP_3_R1 channels through Grp78. In general, APP prevents the Grp78-IP_3_R1 interaction by binding Grp78 and eventually inhibits channel opening. When APP is absent, more Grp78 binds to IP_3_R1, facilitating the passage of calcium ions.

We used a VDAC2 antibody for immunoprecipitation and probed with APP and α-synuclein antibodies by western blotting. FL-APP and α-synuclein were pulled down by the VDAC2 antibody, indicating that the regulatory effects of endogenous FL-APP and α-synuclein on VDAC2 involve interactions between these proteins (Fig. 6E). To further confirm the existence of the IP_3_R1-Grp75-VDAC2 axis in the rat hippocampus, we also performed coimmunoprecipitation with IP_3_R_S_ and VDAC2 antibodies and confirmed the interactions of IP_3_R1-Grp75 (Fig. S3D) and Grp75-VDAC2 (Fig. S3E), but no interactions between APP and SNCA proteins (Fig. S3F).

We concluded that MAM-localized FL-APP prevents IP_3_R1 from releasing calcium into the MAM region by binding to Grp78 and promotes calcium flux through the OMM by interacting with the VDAC2 channel. α-Synuclein binds to VDAC2 and facilitates calcium flux through the OMM into the matrix (Fig. 6F).

### APP and α-synuclein redundantly facilitate mitochondrial calcium outflow

To further assess whether mitochondrial calcium release is affected by *APP* and *SNCA* deficiency, we stimulated dye-loaded hippocampal mitochondria with a series of calcium concentrations from 0.75 mM to 12 mM (Fig. 6G). In the range of 0.75 mM–3 mM, mitochondrial calcium uptake significantly increased. When it reached 3 mM to 6 mM, the curve no longer appeared stepped but tended to be stable, indicating that the mitochondrial buffering capacity had reached the upper limit. This was obvious because the fluorescence count dropped briefly after 6 mM stimulation and quickly returned to the previous plateau level of 3 mM stimulation. The plateau level of 3 mM–6 mM stimulation was 9.18 times that of the initial resting state (Fig. S4A), indicating that the upper limit of *WT* mitochondrial buffering capacity was ~9.18 times that of its resting pool. The upper limit of buffering capacity in *APP KO, SNCA KO* and *DKO* mitochondria showed no significant difference from *WT*, approximatly 9.59, 8.79 and 8.80 times, respectively (Fig. S4B), indicating that *APP* and *SNCA* deficiency does not alter the upper limit of hippocampal mitochondrial buffering capacity.

When extra calcium stimulation was elevated to 12 mM (Fig. 6G), the fluorescence count dropped sharply, suggesting continuous mPTP opening to allow matrix calcium release. *WT* mitochondria released 22.7% of their maximum calcium capacity reached during 3 mM–6 mM stimulation, while *APP KO*, *SNCA KO* and *DKO* mitochondria released less calcium, 13.1%, 12.4% and 13.4% of their maximum calcium capacity level, respectively (Fig. 6H). These results suggest that endogenous APP and α-synuclein redundantly promote mitochondrial calcium outflow.

## Discussion

In this study, we found that knockout of *APP* or *SNCA* leads to neurodegenerative changes in the rat hippocampus, demonstrating that they are intrinsic inhibitors of neurodegeneration. Therefore, we proposed the hypothesis of “chronic impairment of physiological function” that is, injury to *APP* and *SNCA* function is the core pathology of AD and PD in the early stage. Due to their importance in avoiding neurodegeneration, cells ensure *APP* and *SNCA* physiological functions at multiple levels, such as maintaining relatively stable levels of precursors and their cleavages and carrying out correct intracellular sorting and localization. Any changes in the regulation will have local, limited and weak impacts in the short term. In fact, at this time, cell sense damage to these gene functions, initiating compensation and rescue mechanisms, including the expression of new *APP* and *SNCA* genes. However, due to the mutation carried by the coding gene, the newly expressed proteins are still malfunctioning, resulting in a vicious cycle of increasing compensation mechanisms, and finally, irreversible damage signals are released in the form of aggregation of Aβ and α-synuclein, leading to broader intracellular and extracellular responses. This eventually leads to neuronal death and degenerative changes in the central nervous system.

According to our study, the pathology of *APP* and *SNCA* deficiency in hippocampal degeneration converges to the maintenance of mitochondrial calcium homeostasis. APP and α-synuclein collaboratively regulate MAM-mediated mitochondrial calcium inflow from the ER via the IP_3_R1-Grp75-VDAC2 axis and redundantly promote mitochondrial calcium release. We suggest that the severity of hippocampal degeneration is correlated with APP and α-synuclein in regulating MAM-mediated mitochondrial calcium homeostasis. Although α-synuclein promotes mitochondrial calcium uptake and release, mitochondrial calcium overload in the *SNCA*-deficient hippocampus should be primarily due to perturbing mitochondrial calcium release but not uptake. In the *APP*-deficient hippocampus, mitochondria take up more calcium through the MAM and release less calcium, resulting in more severe mitochondrial calcium overload than *SNCA* deficiency. Therefore, the spatial memory of *APP KO* rats is worse than that of *SNCA KO* rats, suggesting that the IP_3_R1-Grp75-VDAC2 axis is a common drug target for the early prevention of AD and PD. However, other reasons should be considered. The compensatory expression of APP in *SNCA KO* hippocampus may generate more sAPPα, which rescue rat memory [40, 41].

The dose complementarity between APP and α-synuclein may be due to their compensation for regulating mitochondrial calcium homeostasis. APP inhibits the entry of excess calcium ions into mitochondria through MAM and promotes mitochondrial calcium release. After deletion, *APP KO* rat had mitochondrial calcium overload. At this point, compensatory expressed α-synuclein will reduce calcium levels by promoting mitochondrial calcium release. When *SNCA* is depleted, the upregulated APP inhibits the flow of ER calcium through the MAM into mitochondria and facilitates calcium efflux, temporarily alleviating the dilemma of ER-mitochondrial calcium homeostasis in the *SNCA KO* hippocampus. Unfortunately, dysfunctions of either *APP* or *SNCA* genes lead to both ER stress and mitochondrial dysfunction, worsening the intracellular environment in the direction of promoting protein precipitation, and this dose-compensation effect may accelerate abnormal aggregation.

Admittedly, some limitations were present in our research. First, the analysis of mitochondrial sensitivity to calcium channel antagonists and immunoprecipitation revealed that APP and α-synuclein controlled calcium flux through the IP_3_R1-Grp75-VDAC2 axis, but the involvement of VDAC1 could not be excluded. The antagonist NSC15364 has been proven to inhibit VDAC1 oligomerization and apoptosis but not calcium conductance based on our study (Fig. S3A). The VDAC1-VDAC2 interaction (Fig. S3E) suggests the possibility that VDAC1 might bind to VDAC2 to participate in APP- and α-synuclein-mediated MAM calcium regulation. Second, ORAI1 upregulation and elevated PDH^s293^ phosphorylation indicated that *APP* or *SNCA* deficiency caused mitochondrial calcium overload during ER calcium depletion, but in vivo measurement of calcium levels in the ER lumen and mitochondrial matrix would be more convincing.

In previous studies, the roles of *APP* and *SNCA* in the pathology of AD and PD were mostly studied separately. The phenotype in *APP*-null mice supports the critical role of APP in maintaining mitochondrial homeostasis and cognitive ability [42, 43], which was also demonstrated in our study of *APP KO* rats. The mitochondrial localization details of endogenous APP are helpful in understanding early-AD pathology but remain obscure. A widely cited study indicated that mutated but not wild-type APP blocked mitochondrial TOM40/TIM23 channels in the human brain [8]. Subsequent studies have described the mitochondrial localization of APP in the postmortem brain of normal control humans [44, 45]. A variety of APP processing enzymes and products that were found in mitochondria [45–47] are indirect evidences. According to our data, APP is rarely present in pure mitochondria but is enriched in the MAM fraction isolated from the *WT* hippocampus (Fig. 3E), suggesting that MAM may be the primary subregion for APP to regulate mitochondria under normal conditions.

MAM deposition of the APP β-cleavage product C99 has been shown to decrease mitochondrial respiration [12]. Aβ generated in MAMs by γ-cleavage of C99 increases the MAM content and mitochondrial calcium level [48, 49]. These results suggest that APP might also be related to MAM-mediated calcium regulation. Cultured astrocytes from *APP KO* mice showed that *APP* depletion reduced mitochondrial calcium waves after external ATP stimulation [43]. Based on our study, mitochondria isolated from the hippocampus of *APP KO* rats reserved more calcium than those of *WT* (Fig. 5C–F, Fig. 6G, H), which was due to the bidirectional control of MAM calcium flow through IP_3_R1 and VDAC2 (Fig. 6C, E). Thus, we suggest that the effect of *APP* deficiency on mitochondrial calcium in neurons and astrocytes may be different. An in vitro study of α-synuclein found partial blocking of the VDAC pore and mentioned it as a potent regulator of VDAC-facilitated calcium transport [50]. We provided evidence that α-synuclein promoted mitochondrial calcium entry and outflow in the hippocampus via VDAC2 at physiological concentrations, suggesting that a considerable abundance of α-synuclein in hippocampal cells is necessary for maintaining mitochondrial calcium homeostasis under normal physiological conditions but not only in pathological conditions. A detailed description of MAM-localized APP and α-synuclein is shown in Fig. 6F, which is similar to other group [51].

Both APP and α-synuclein are located on the MAM structure, suggesting that both are closely related to MAM-mediated physiological functions. Our study explored their correlations on MAM-mediated calcium homeostasis and found their dose-compensatory relationship, providing their converged regulation axis IP_3_R1-Grp75-VDAC2 as the common drug target for AD and PD.

## Materials and methods

### Generation of gene knockout rats by TALEN

Knockout rat lines were generated through TALEN technology from K&D Gene Technology (Wuhan, China). *APP* gene exon 3 and *SNCA* gene exon 1 in Sprague Dawley (SD) rats were the TALEN targets. The active TALEN was transcribed into mRNA in vitro and then injected into fertilized eggs of SD rats. The targeted embryos were transferred into the fallopian tubes of the surrogate mother rat. After 21–23 days of gestation, the F_0_ generation was born, and genotyping was performed using the primers listed in Table S1 to identify chimeric rats. Chimeric offspring were collected and tested for germline transmission by crossing with *WT* rats for at least five generations. Then, the heterozygotes were crossed to produce homozygotes.

### Rat husbandry and treatment

The animals were housed in the Animal Biosafety Level 3 Laboratory of Wuhan University with the approval of the Institutional Animal Care and Use Committee. All rats were fed a standard diet and exposed to light for 12 h per day at a fixed time. Male rats aged 3 months were grouped by genotypes. The animals in the group were randomly allocated. Rats were decapitated and dissected for sample collection. The rats were intraperitoneally injected with 2% pentobarbital at a dose of 0.5 mL/0.1 kg of body weight for anesthesia.

### Antibodies and reagents

The following antibodies were used for immunoblotting: APP (#D260097-0200, BBI, 1:1000), SNCA (#A20407, ABclonal, 1:1000), β-actin (#AC026, ABclonal, 1:100000), Hsp60 (#15282-1-AP, Proteintech, 1:1000), Flot1 (#15571-1-AP, Proteintech, 1:1000), MFN2 (#12186-1-AP, Proteintech, 1:1000), VDAC1 (#55259-1-AP, Proteintech, 1:1000), VDAC2 (#11663-1-AP, Proteintech, 1:1000), VAPB (#14477-1-AP, Proteintech, 1:1000), Drp1 (#12957-1-AP, Proteintech, 1:1000), LC3-B (#A19665, ABclonal, 1:1000), OPA1 (#ab42364, Abcam, 1:1000), Grp78 (#11587-1-AP, Proteintech, 1:1000), ATF4 (#sc-390063, Santa Cruz Biotechnology, 1:100), CHOP (#sc-37351, Santa Cruz Biotechnology, 1:100), Caspase-3 (#sc-56053, Santa Cruz Biotechnology, 1:100), IP_3_Rs (#sc-377518, Santa Cruz Biotechnology, 1:100), RyR1 (#E-AB-13584, Elabscience, 1:400), COX4 (#11242-1-AP, Proteintech, 1:1000), Cyt C (#10993-1-AP, Proteintech, 1:1000), Grp75 (#14887-1-AP, Proteintech, 1:1000), ORAI1 (#66223-1-IG, Proteintech, 1:1000), PDHA (#A1895, ABclonal, 1:1000), and PDHA^S293^ (#AP1022, ABclonal, 1:1000). The following antibody was used for immunofluorescence: NeuN (#3075598, Sigma, 1:800).

The following antagonists were used for calcium channel inhibition: 2-aminoethyl diphenylborinate (2-APB, #HY-W009724, MCE), azumolene (#HY-113920A, MCE), erastin (#HY-15763, MCE) and NSC15364 (#HY-108937, MCE). The calcium ion fluorescent probe Fluo-4 AM (#S1060, Beyotime) was used to detect mitochondrial calcium according to the manufacturer’s instructions.

### TEM

Hippocampal tissues were dissected from 3-month-old male rats and fixed in cold 2.5% glutaraldehyde overnight. Then, the tissues were postfixed in 1% osmium tetroxide, contrasted with 1% uranyl acetate, dehydrated in increasing ethanol concentrations and embedded in Durcupan. The tissue blocks were immersed in liquid nitrogen to release the coverslips and then sliced into ultrathin sections. The sections were analyzed with a transmission electron microscope (HT7700, Hitachi). ImageJ was used in the quantification of TEM photos.

### Subcellular fractionation of the ER, MAM, crude and pure mitochondria from hippocampal tissues

Subcellular fractionation was performed as described in a previously reported protocol [13, 23]. All steps were performed at 4 °C or on ice. Dissected hippocampal tissues from 6 male rat brains were mixed and cut into pieces in homogenization buffer (225 mM mannitol, 75 mM sucrose, 30 mM Tris-HCl (pH 7.4), and 0.1 mM EGTA). The tissues were homogenized and centrifuged at 500 × g twice for 5 min each to obtain the supernatant. The supernatant was centrifuged at 10,300 × g for 10 min to separate the pellet (P1, Mc fraction) and the resultant supernatant (S1). For ER isolation, the resultant supernatant (S1) was centrifuged at 20,000 × g for 30 min. The pellet consisted of the lysosomal and PM fractions (P2). The supernatant (S2) was then centrifuged at 100,000 × g for 90 min (P40ST rotor; Hitachi, Japan) to separate the pellet (P3, ER fraction) and supernatant (S3, cytosolic fraction).

For MAM and pure mitochondria (Mp fraction) isolation, crude mitochondria (P1, Mc fraction) were resuspended in MAM isolation buffer (250 mM mannitol, 5 mM HEPES (pH 7.4) and 0.5 mM EGTA), loaded on a 30% v/v Percoll gradient (Percoll medium: 225 mM mannitol, 25 mM HEPES (pH 7.4), and 1 mM EGTA) in a 12-ml ultracentrifuge tube, and then centrifuged at 95,000 × g for 30 min (P40ST rotor; Hitachi, Japan). Two dense bands were identified, i.e., an upper MAM band (MAM fraction) and lower pure mitochondria band (Mp fraction). The MAM band was isolated, resuspended, and further centrifuged at 100,000 × g for 90 min (P40ST rotor, Hitachi, Japan) to pellet the MAM fraction. The Mp band was isolated, resuspended, and centrifuged at 6,300 × g for 10 min to pellet the Mp fraction.

### Isolation of crude mitochondria from the rat hippocampus

Mitochondria were isolated at 4 °C or on ice. Hippocampal tissues were dissected and minced in mitochondrial isolation buffer (225 mM mannitol, 75 mM sucrose, 5 mM MOPS, and 2 mM taurine (pH 7.25)). The tissues were homogenized in a Teflon glass homogenizer at 1,800 rpm for 20 strokes. The mixture was centrifuged for 5 min at 500 × g to collect the supernatant and centrifuged again. The supernatant was then centrifuged at 10,300 × g for 10 min to pellet the mitochondria and washed three times at 6,300 × g for 10 min each. As much of the supernatant was removed as possible, and the isolated mitochondrial samples were weighed with an analytical balance. The mitochondria were resuspended to a final concentration of 0.1 mg/μL in mitochondrial isolation buffer for follow-up experiments. Ten microliters of resuspended mitochondria were used for protein content analysis using a Detergent Compatible Bradford Protein Assay Kit (Beyotime).

### Mitochondrial calcium uptake assay

The assay was performed as described in a previously reported protocol [52]. For loading of the mitochondrial calcium-sensitive dye Fluo 4-AM, resuspended mitochondria were mixed with an equal volume of diluted Fluo 4-AM dye (4 μM in mitochondrial isolation buffer) and incubated at 28 °C for 30 min. For antagonist treatment, mitochondria were first loaded with Fluo 4-AM dye for 30 min, antagonist working solution was added, and the mitochondria were incubated at 28 °C for another 30 min. Then, the stained mitochondria were washed three times and resuspended at a concentration of 0.1 mg/μL in detection buffer (137 mM KCl, 20 μM EGTA, 20 mM HEPES, 5 mM glutamate, 5 mM malate, 2 mM KH_2_PO_4_, 5 mM NaCl (pH 7.15); 10 μM CGP37157 and 2.5 μM thapsigargin were added before use). Ten milligrams of stained mitochondria in 100 μL of detection buffer were used for a single assay. Extra stimulation buffer was prepared using CaCl_2_, which was dissolved in detection buffer as well. Calcium was added in a volume of 20 μL each time. Fluorescence was measured with a multiscan spectrophotometer (SPECTRAMAX i3X, Molecular Devices) at 25 °C.

### Mitochondrial resting matrix calcium measurement

The assay was performed as described in a previously reported protocol [52]. Mitochondria were isolated in mitochondrial isolation buffer without EGTA. Mitochondria (10 mg) were pelleted and diluted with 0.6 mol/L HCl (1:10 wt/vol). The resuspended mitochondria were sonicated at a fixed output of 20 s 3 times at 4 °C (Bioruptor). The mixture was heated at 95 °C for 30 min and centrifuged at 13,000 × g for 10 min to obtain the supernatant. The supernatant was neutralized with 0.6 mol/L NaOH, and the calcium content was measured with an o-cresolphthalein complexone calcium assay kit (Beyotime).

### Western blotting and immunoprecipitation

Proteins were extracted by incubation with western blot and IP lysis buffer (Beyotime Biotechnology) supplemented with complete protease inhibitor (MCE) and phosphatase inhibitor (YEASEN Biotechnology) for 60 min on ice. After centrifugation at 13,000 × g for 10 min at 4 °C, the supernatants were collected. Equal amounts of protein samples were mixed with loading buffer and heated at 100 °C for 10 min. The denatured protein samples were separated by electrophoresis on a 6 ~ 15% SDS–PAGE gel and transferred onto polyvinylidene fluoride (PVDF) membranes. After blocking for 1 h with 5% nonfat milk, the membranes were immunoblotted with the indicated primary antibodies overnight at 4 °C. After washing three times in TBST, the membranes were incubated with an appropriate anti-mouse or anti-rabbit secondary antibody, and the signals were detected with an enhanced chemiluminescence (ECL) kit.

Immunoprecipitation was carried out using Protein A/G magnetic beads (MCE) following the manufacturer’s instructions. Rat hippocampal tissue was homogenized in RIPA lysis buffer (Beyotime) with proteinase cocktail inhibitor (MCE) and phosphatase inhibitor (YEASEN Biotechnology). Primary antibodies or normal control IgG were incubated with 40 μL beads solubilized in PBS buffer containing 0.3% Triton X-100 (PBST) at 4 °C for 10 h. The prepared hippocampal lysates were incubated with the antibody-coated beads overnight at 4 °C. After incubation, the beads were washed four times with PBST buffer. The proteins were eluted from the beads by heating in 1 × SDS–PAGE loading buffer and analyzed by western blotting.

### DNA/RNA isolation, qRT–PCR and mitochondrial content assay

Hippocampal tissues were dissected from the brains of 3-month-old male rats and immediately immersed in lysis buffer. DNA was isolated through the phenol chloroform extraction method, and total RNA was extracted through the TRIzol method. Reverse transcription was performed with a reverse transcription kit (TIANGEN Company, China) at 42 °C for 15 min and then 95 °C for 3 min.

Real-time quantitative polymerase chain reaction (qRT–PCR) was used to analyze the transcription levels of *GAPDH*, *GRP78*, *EIF2S1*, *ATF4*, *ATF6*, *XBP1S*, *CHOP*, *BAX* and *BCL2*. The primers used for qRT–PCR are shown in Table S1. qRT–PCR was performed on a quantitative PCR detection system (Bio-Rad) with Fast Start Universal SYBR Green Master Mix (Vazyme) under the following conditions: 5 min at 95 °C, 10 s at 95 °C, 35 s at 60 °C, and 10 s at 65 °C. Melting curve data were collected to verify the specificity of PCR, and each gene was analyzed in triplicate. The relative gene expression levels were calculated using the 2^-ΔΔCt^ method.

For the mitochondrial content assay, *mt-Nd1* and *Cftr* gene levels relative to total DNA levels were evaluated. The relative gene expression levels were calculated using the 2^-ΔΔCt^ method.

### Immunofluorescence and Nissl staining

Rats were perfused with 4% paraformaldehyde under deep anesthesia, and the animal brains were dissected after perfusion. The brains were then fixed overnight at 4 °C and dehydrated in 10%, 20%, and 30% sucrose/PBS. Brain sections with a thickness of 15 µm were obtained. The tissues were washed with PBS for 5 min at RT, blocked with 5% goat serum and 0.2% Triton-X/PBS for 1.5 h at RT and incubated with primary antibodies at 4 °C overnight. After washing with PBS, the slices were stained with dye-labeled secondary antibodies for 2 h at RT. The slices were mounted in media with DAPI (VECTASHIELD), and the hippocampal CA1 region was observed under 60× objectives with a Nikon A1 confocal microscope.

For Nissl’s staining, cryostat sections were stained with cresol purple at RT for 30 min, washed and mounted with neutral resin. The hippocampal CA1 region was observed under a microscope.

### Mitochondrial ATP and serum lactate level assay

Isolated hippocampal mitochondria were lysed and analyzed using a Luminescent ATP Detection Test Kit (Beyotime) according to the manufacturer’s instructions. Blood was collected via cardiac puncture. Lactate levels in the serum were measured according to the manufacturer’s instructions using a lactate assay kit (Nanjing Jiancheng Bioengineering Institute).

### MWM test

The MWM test was performed as previously described [15]. A total of 10 3-month-old male rats per genotype were weighed, and rats with similar body weights were selected for the experiment. The experiment was conducted in a quiet and isolated environment that was familiar to all rats. The rats were trained in 4 trials per day for 5 days according to the protocol and were tested on the 6th day after the escape platform was removed. Videos were recorded and analyzed with Smart 3.0 software (Panlab).

### Statistical analysis

GraphPad Prism 7.0 was used for statistical analysis. Data were from at least three independent experiments with at least three rats per genotype in each assay. Data of individual rats are indicated by dots in the charts. Data collection and analysis were executed blindly between investigators and analyzers. The data are expressed as the mean ± SEM and were analyzed using Student’s *t*-test and one-way ANOVA as indicated. Differences are indicated by asterisks (0.01 < *p < 0.05, **p < 0.01 and ***p < 0.001 indicate significant differences, *n.s*. indicates no significance).

## Date availability

All data generated and analyzed during this study are included in this article and its extended data are available from the corresponding author on reasonable request.

## Supplementary information

supplementary information

AJ-checklist

origianl western blots
