# Supplementary material for: Pathological convergence of APP and SNCA deficiency in hippocampal degeneration of young rats
Source: Cell Death Dis. 2023 May 13;14(5):325. doi: 10.1038/s41419-023-05846-5 (PMC10183039; doi:10.1038/s41419-023-05846-5)
Supplement: Supplementary file 1 — supplementary information [file 41419_2023_5846_MOESM1_ESM.docx]

# **Supplementary material for**:

**Pathological convergence of *APP* and *SNCA* deficiency in hippocampal degeneration of young rats**

Yajie Wang^1#^, Zhikang Miao^1#^, Chang Xu^1,2^, Ying Cai^1^, Yuting Yang^1^, Yue Hu^1^, Mengna Zhao^1,3^, Yue Shao^1^, Zhiqiang Li^1^, Jincao Chen^1^, Shi Chen^1,3*^, Lianrong Wang^1,2*^

# These authors contributed equally

Correspondence to: Lianrong Wang, Shi Chen.

1. mail: lianrong@whu.edu.cn, shichen@whu.edu.cn.

This PDF file includes:

Figures S1 to S4

Tables S1

**
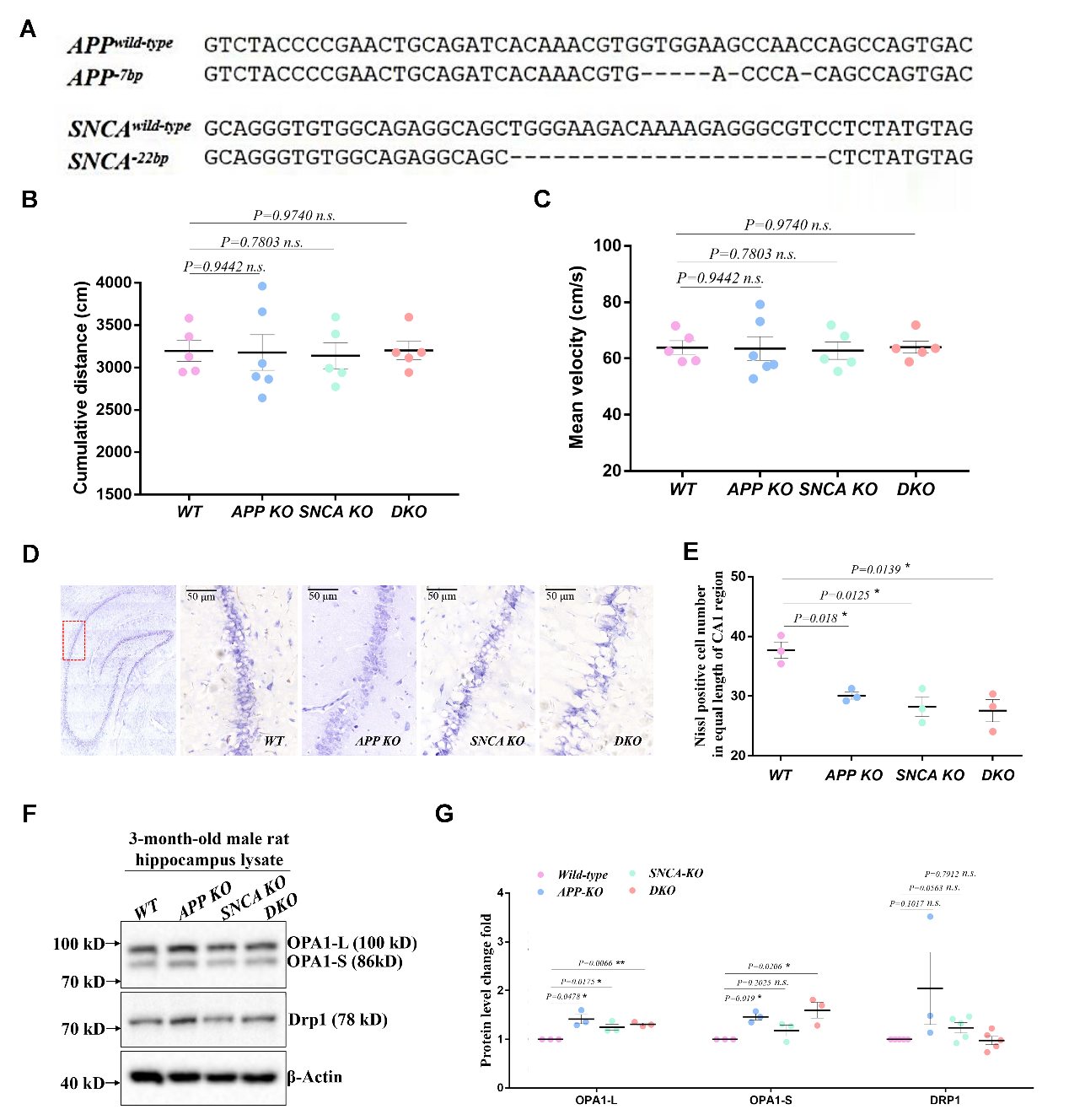
**

**Figure S1**. **Hippocampal degeneration appears in young *APP KO* and *SNCA KO* rats**. (**A**) Genotyping of *APP KO* and *SNCA KO* rats. Rats with 7 bp deletions in exon 3 of the *APP* gene and 22 bp deletions in exon 2 of the *SNCA* gene were reserved for homozygous breeding. These deletions cause frameshift mutations. (**B-C**) Cumulative distance and mean velocity of the rats in the MWM probe test. The data of individual rats are indicated by dots, and the mean ± SEM for each genotype is presented. T tests were used, and *n.s.* indicates significant differences. (**D-E**) Nissl staining in the rat hippocampus. Pyramidal neurons in the hippocampal CA1 region (indicated by the red box) were counted. Three rats of each genotype were studied. The data are presented in **E**) as the mean ± SEM. T tests were performed. *p<0.05 indicates significant differences. **(F-G)** The protein levels of mitochondrial fission and fusion regulators were altered in the hippocampi of 3-month-old male rats. Three independent experiments including three rats of each genotype were performed. The data are shown as the mean ± SEM in the chart in **E**). One-way ANOVA was performed. *p<0.05, **p<0.01 indicate significant differences, and *n.s.* indicates no significance.

**
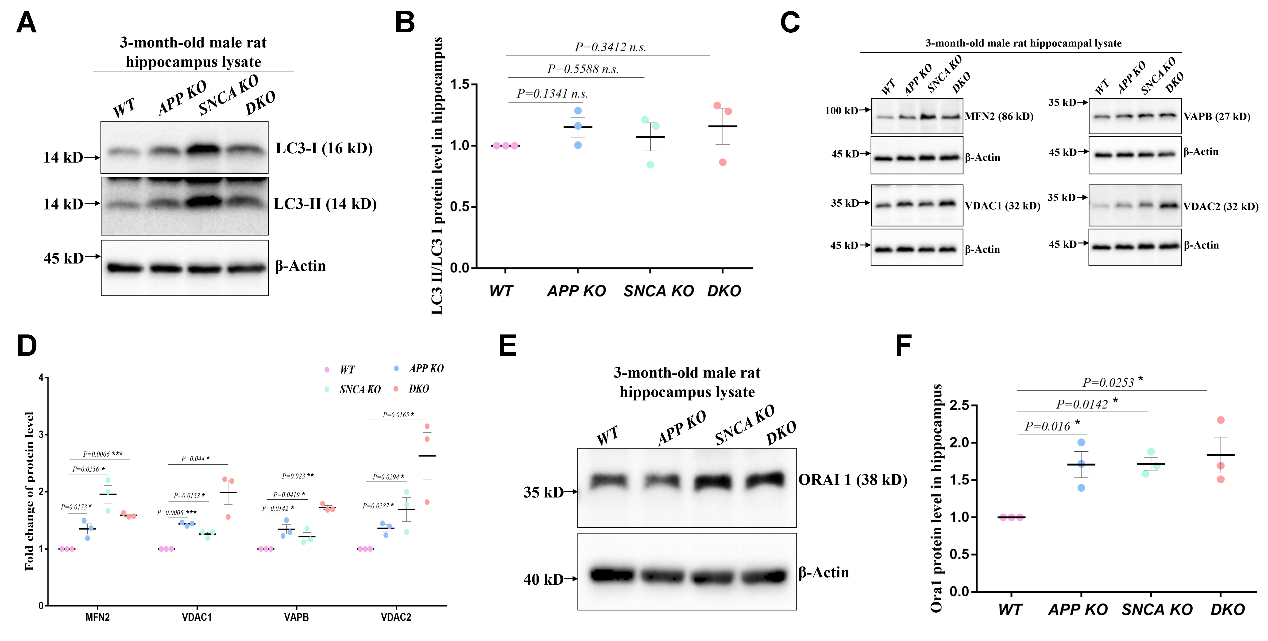
**

**Figure S2**. **Mitochondrial and ER abnormalities in young *APP KO* and *SNCA KO* rats**. (**A-B**) The expression of the mitophagy marker LC-II in hippocampal lysates from *WT*, *APP KO*, *SNCA KO* and *DKO* rats was measured. Three 3-month-old male rats per genotype were studied, and the data are shown as the mean ± SEM in the chart in **B**). One-way ANOVA was performed. *n.s.* indicates no significance. (**C-D**) The protein levels of MAM tethers in hippocampal lysates from 3-month-old male *WT*, *APP KO*, *SNCA* *KO* and *DKO* rats were measured. Three independent experiments including 3 rats per genotype were performed. The data are shown as the mean ± SEM in **D**). T tests were used. *p<0.05 and **p<0.01 indicate significant differences. (**E-F**) The protein level of the SOCE-related channel OraI1 in hippocampal lysates from *WT*, *APP KO*, *SNCA KO* and *DKO* rats was measured. Three 3-month-old male rats per genotype were studied, and the data are shown as the mean ± SEM in the chart in **F**). One-way ANOVA was performed. *p<0.05 indicates significant differences.

**
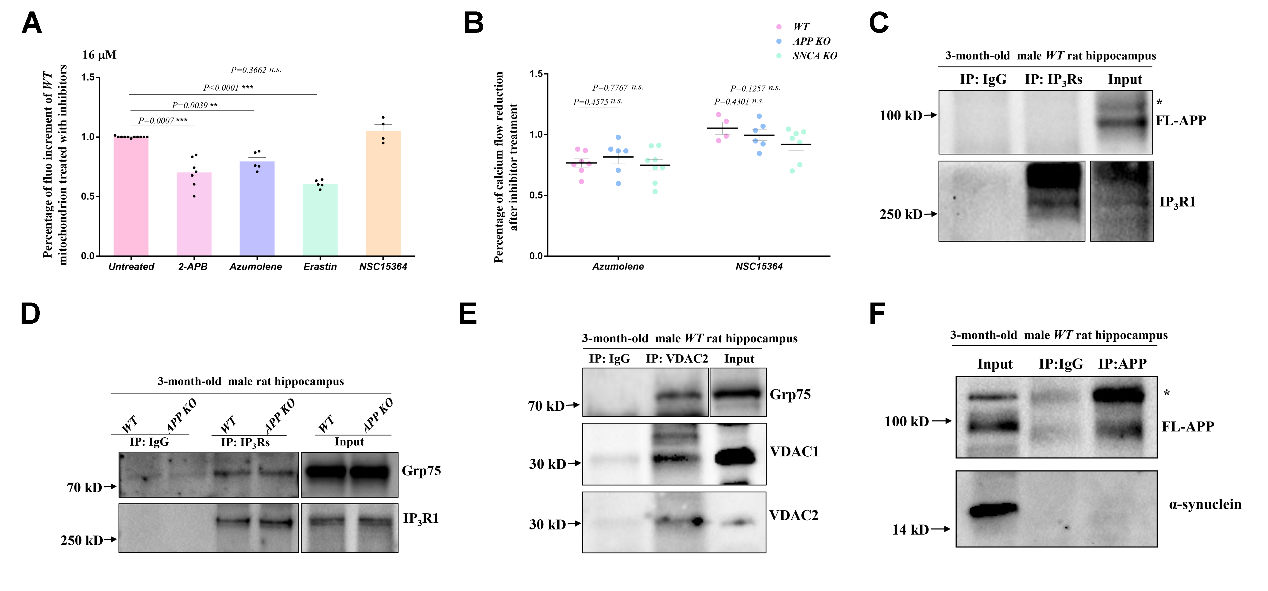
**

**Figure S3**. **APP and α-synuclein regulate calcium flux through the IP_3_R1-Grp75-VDAC2 axis**. (**A**) Sensitivity of *WT* mitochondria to calcium channel antagonists. The percent of net fluorescence increase in 10 mg crude mitochondria treated with inhibitors relative to the untreated *WT* control was measured upon 16 μM calcium stimulation. Mitochondria were isolated from the hippocampi of 3-month-old male rats, and three independent experiments including at least 4 *WT* rats were performed. The data of individual rats are indicated by dots, and the mean ± SEM for each antagonist treatment is presented. T tests were performed. **p<0.01 and ***p<0.001 indicate significant differences, and *n.s.* indicates no significance. (**B**) The crude mitochondria from *APP KO* and *SNCA KO* hippocampi were not sensitive to azumolene or NSC15364. The percentages of net fluorescence increased in the antagonist-treated mitochondria relative to their untreated controls were determined after 16 μM calcium stimulation. Ten milligrams of crude mitochondria isolated from the hippocampi of 3-month-old male rats were used for the assay, and three independent experiments including at least 4 rats per genotype were performed. The data of individual rats are indicated by dots, and the mean ± SEM for each genotype is presented. T tests were performed. *n.s.* indicates no significance. (**C**) No interaction between FL-APP and IP_3_Rs in the hippocampal lysate of 3-month-old male *WT* rats. An anti-IP_3_Rs antibody was used for immunoprecipitation, and an anti-APP antibody was used for western blotting. The asterisk indicates a stray band. (**D**) Interaction between IP_3_R1 and Grp75 in the hippocampal lysate of 3-month-old male *WT* rats. An anti-IP_3_Rs antibody was used for immunoprecipitation, and IP_3_R1 protein pulled down from the lysate was used as the loading control. Grp75 levels were measured by western blotting. (**E)** Interactions of VDAC2 with VDAC1 and Grp75 in the hippocampal lysate of 3-month-old male *WT* rats. An anti-VDAC2 antibody was used for immunoprecipitation, and anti-Grp75 and anti-VDAC1 antibodies were used for western blotting. Three independent experiments including 3 rats per genotype were performed. (**F)** No interaction between FL-APP and α-synuclein in the hippocampal lysate of 3-month-old male WT rats. An anti-APP antibody was used for immunoprecipitation, and an anti-α-synuclein antibody was used for western blotting. The asterisk indicates a stray band.


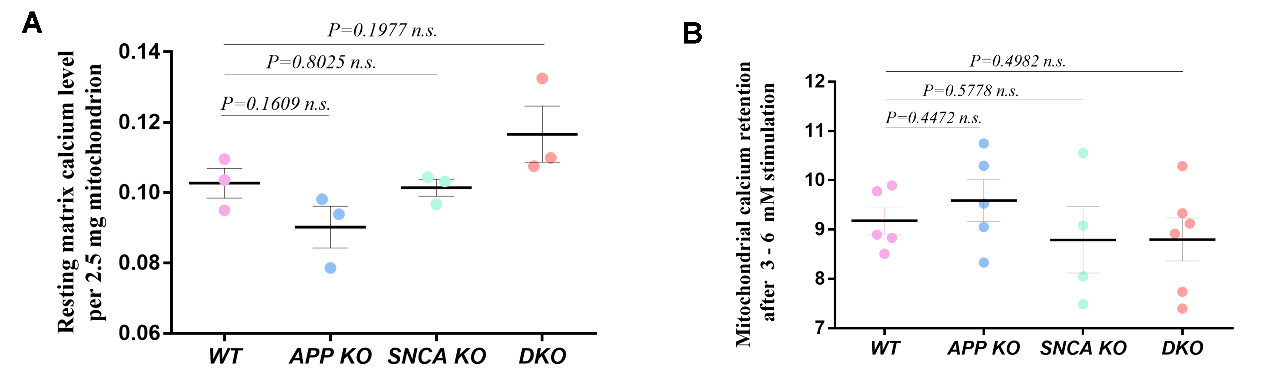


**Figure S4**. **Resting matrix calcium level and maximal calcium retention of the mitochondria isolated from *APP KO* and *SNCA KO* hippocampi**. (**A**) Resting matrix calcium levels in mitochondria isolated from the hippocampi of 3-month-old male rats of the indicated genotypes. Three independent experiments were performed and the data of individual rats are indicated by dots. The data are shown as the mean ± SEM. T tests were performed. *n.s.* indicates no significance. (**B**) Maximal mitochondrial calcium buffering capacity was measured. The average F_1_/F_0_ value after 3-6 mM calcium stimulation in Figure 6G was used as an index of maximal mitochondrial calcium buffering capacity. At least four male rats of 3-month-old per genotype were included in three independent experiments. The data of individual rats are indicated by dots, and the mean ± SEM for each genotype is presented. T tests were used for statistical analysis. *n.s.* indicates no significance.

| Name | Sequence | Purpose |
| --- | --- | --- |
| rat-mt-Nd1-Forward | 5’-ACCTCACCCCCTTATCAACC-3’ | Mitochondrial |
| rat-mt-Nd1-Reverse | 5’-AGGGCTCCGAATAGGGAGTA-3’ | content assay |
| rat-Cftr-Forward | 5’-GCTAGGTTCAGCCTCCACAG-3’ |  |
| rat-Cftr-Reverse | 5’-CAGCCAAACATCAAGCAGAA-3’ |  |
| Eif2s1-Forward | 5’-GCTTGCTATGGTTACGAAGGC-3’ | RT–qPCR |
| Eif2s1-Reverse | 5’-CATCACATACCTGGGTGGAG-3’ | analysis for |
| ATF4-Forward | 5’-TCTGTATGAGCCCTGAGTCCTACCT-3’ | ER stress |
| ATF4-Reverse | 5’-GGTCATAAGGTTTGGGTCGAGAACCAC-3’ | related gene |
| CHOP-Forward | 5’-CCTGAAAGCAGAAACCGGTC-3’ |  |
| CHOP-Reverse | 5’-CCTCATACCAGGCTTCCAGC-3’ |  |
| Xbp1s-Forward | 5’-GAGTCCGCAGCAGGTGC-3’ |  |
| Xbp1s-Reverse | 5’-GGTCCAACTTGTCCAGAATGC-3’ |  |
| ATF6-Forward | 5’-CGAGGGAGAGGTGTCTGTTTC-3’ |  |
| ATF6-Reverse | 5’-GTCTTCACCTGGTCCATGAGG-3’ |  |
| GRP78-Forward | 5’-CCTATTCCTGCGTCGGTGTATT-3’ |  |
| GRP78-Reverse | 5’-GGTTGGACGTGAGTTGGTTC-3’ |  |
| Bcl-2-Forward | 5’-TCATGTGTGTGGAGAGCGTC-3’ |  |
| Bcl-2-Reverse | 5’-AGTTCCACAAAGGCATCCCAG-3’ |  |
| Bax-Forward | 5’-GCACGTCTGCGGGGAGT-3’ |  |
| Bax-Reverse | 5’-TTCTTGGTGGATGCGTCCTG-3’ |  |
| GAPDH-Forward | 5’-GGAAAGCTGTGGCGTGAT-3’ |  |
| GAPDH-Reverse | 5’-AAGGTGGAAGAATGGGAGTT-3’ |  |
| APP-Forward | 5’-TGGTGATGCTGAAGAA-3’ | Rat |
| APP-Reverse | 5’-TGGTGATGCTGAAGAA-3’ | genotyping |
| SNCA-Forward | 5’-TGGTGATGCTGAAGAA-3’ |  |
| SNCA-Reverse | 5’-TGGTGATGCTGAAGAA-3’ |  |

**Supplementary table S1**. **Primer sequences used for genotyping and qPCR**
